# Two-Year Outcomes of Prostatic Artery Embolization for Symptomatic Benign Prostatic Hyperplasia: An International, Multicenter, Prospective Study

**DOI:** 10.1007/s00270-024-03802-0

**Published:** 2024-09-04

**Authors:** Marc R. Sapoval, Shivank Bhatia, Carole Déan, Antonio Rampoldi, Francisco César Carnevale, Clare Bent, Charles R. Tapping, Simone Bongiovanni, Jeremy Taylor, Jayson S. Brower, Michael Rush, Justin P. McWilliams, Mark W. Little, the PROstate Study Investigators, Olivier Pellerin, Fabiane Barbosa, Peyman Borghei, Greg E. Gin, Edward Uchio, Adam N. Plotnik, James H. Briggs, Andrew MacDonald, Srini Tummala, Hemendra Navinchandra Shah, Issam M. Kably, Keith Pereira, James Katrivesis, Keng Lim Ng, Kirubahara Vaheesan, Mina Behdad, Sarah MacGill, Sarah Crosbie, Madita Gavrila, Susan Anthony, Lia Quezada, Ricardo Aleman, Cynthia Toot Ferguson, Far Ahmed-Timms, Alexandra Edwards

**Affiliations:** 1 Department of Vascular and Oncological Interventional Radiology, Assistance Publique-Hôpitaux de Paris, Hôpital Européen Georges Pompidou, 20 Rue Leblanc, 75015 Paris, France; 2 Department of Interventional Radiology, Miller School of Medicine, University of Miami, Miami, FL USA; 3 Department of Interventional Radiology, Ospedale Niguarda Ca’ Granda, Milan, Italy; 4 Department of Radiology, Instituto de Radiologia do Hospital das Clínicas da Faculdade de Medicina da Universidade de São Paulo, São Paulo, SP Brazil; 5 Department of Interventional Radiology, Royal Bournemouth and Christchurch Hospital, Bournemouth, UK; 6 Department of Radiology, The Churchill Hospital, Oxford, UK; 7 Department of Radiology, Azienda Ospedaliera S. Croce E Carle, Cuneo, Italy; 8 Department of Interventional Radiology, Frimley Park Hospital, Surrey, UK; 9 Department of Radiology, Providence Sacred Heart, Spokane, WA USA; 10 Holy Cross South Florida Medical Imaging, Fort Lauderdale, FL USA; 11 Division of Interventional Radiology, Department of Radiology, David Geffen School of Medicine at UCLA, Los Angeles, CA USA; 12 University Department of Radiology, Royal Berkshire Hospital, Reading, UK

**Keywords:** Acute urinary retention, Benign prostatic hyperplasia, Embosphere® Microspheres, International Prostate Symptom Score (IPSS), Lower urinary tract symptoms, Prostatic artery embolization, Quality of life, Sexual Health Inventory for Men (SHIM)

## Abstract

**Purpose:**

To describe clinical outcomes among patients with benign prostatic hyperplasia (BPH) 24 months following prostatic artery embolization (PAE).

**Materials and Methods:**

This was an international, multicenter, prospective trial of males with BPH with lower urinary tract symptoms (LUTS) or acute urinary retention (AUR) treated with PAE. The primary outcome was the 12 month change in the International Prostate Symptom Score (IPSS) for patients referred for bothersome LUTS, or urinary catheter independence for patients treated for AUR. Secondary outcome measures included changes in IPSS at 3 and 24 months, changes in quality of life (QoL), changes in the Sexual Health Inventory for Men (SHIM) questionnaire, technical success rate, and adverse events (AEs). Data were summarized using descriptive statistics.

**Results:**

Four hundred seventy-eight consecutive patients underwent PAE (bothersome LUTS: N = 405; AUR: N = 73), mean age was 70 years. For patients treated for bothersome LUTS, mean total IPSS at baseline was 21.8 and decreased to 9.3, 10.6, and 11.2 at 3, 12, and 24 months following PAE, respectively (all *p* < 0.001); QoL at baseline was 4.7 and decreased to 2.0, 2.1, and 2.3 at 3, 12, and 24 months, respectively (all *p* < 0.001). The mean SHIM score at baseline and 12 months following PAE was 13.8 and 13.9, respectively. Of the 73 patients treated for AUR, 48 (65.8%) had their indwelling catheter removed within 3 months of PAE and remained catheter free at 24 months. Fifty-five patients (11.5%) experienced ≥ 1 AE and 10 (2.1%) experienced a serious AE.

**Conclusion:**

PAE is a safe and effective treatment for symptomatic BPH and LUTS.

*Level of Evidence* Level 3

*Trial registration* ClinicalTrials.gov NCT03527589.

**Graphical Abstract:**

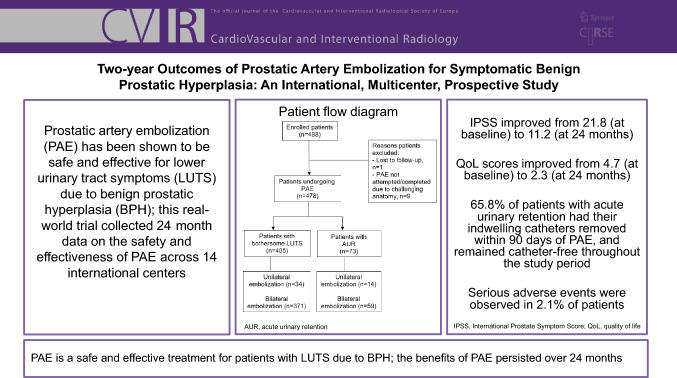

## Introduction

Benign prostatic hyperplasia (BPH) is a common urological condition that affects men [1]. For patients unresponsive to lifestyle modifications and pharmacotherapy, guidelines recommend surgical treatment [2]. Transurethral resection of the prostate (TURP) is the standard surgical therapy for BPH [3]; however, newer surgical therapies (e.g., Urolift, Rezum) have shown promise as minimally invasive treatments that can help avoid complications (e.g., bleeding, incontinence, sexual dysfunction associated with TURP) [2]. Prostatic artery embolization (PAE) is a newer minimally invasive technique that causes partial ischemic necrosis of the prostate gland and softening of the gland that can lead to reduction in BPH and symptomatic improvement [4, 5].

Several clinical trials, cohort studies, reviews, and meta-analyses evaluating clinical outcomes following PAE and other therapies for BPH with lower urinary tract symptoms (LUTS) have been published [6–28]. A common finding in the clinical trial setting is that PAE is an effective therapy that is associated with a generally high rate of technical success. Among trials comparing outcomes following PAE versus TURP, patients that underwent embolization typically experienced similar clinical improvement as those treated with TURP but with fewer adverse events (AEs) [6, 8, 10, 27, 28]. In clinical practice, PAE is considered to be a viable alternative for BPH management, particularly among patients that are unable or refuse surgery [15, 16, 18–20, 29].

As several prior studies have highlighted the safety and effectiveness of PAE, the goal of this study was to provide additional real-world evidence describing clinical outcomes following the procedure. An important aspect of this study was that it captured the safety and effectiveness profiles of PAE across multiple international centers and operators over 24 months.

## Methods

### Study Design

This was a prospective cohort registry study conducted across 14 centers in France, Italy, the United Kingdom, and the United States.

The inclusion criteria included patients over 18 years of age with symptomatic BPH or acute urinary retention (AUR), who were willing to undergo PAE and provided informed consent. Patients were excluded if they were unable or unwilling to provide follow-up information, were undergoing PAE for reasons unrelated to symptomatic LUTS due to BPH, or any other reason the investigator deemed cause for exclusion (e.g. significant comorbidities preventing the patient from lying flat and still). The criteria for inclusion in the study were broad to capture a range of outcomes among a diverse patient population.

### Study Cohorts

This study included two patient cohorts: those with BPH-related bothersome LUTS but without an indwelling bladder catheter (LUTS cohort) and those with AUR due to underlying BPH with a urinary bladder catheter (AUR cohort).

### *PAE* Procedure

All procedures were performed via femoral or radial access according to standard practices at each participating center. In general, the microcatheter was advanced into the prostatic artery using a road mapping technique. The prostatic artery was embolized using 100–300 µm or 300–500 µm Embosphere® Microspheres (Merit Medical, South Jordan, Utah, USA) until total arterial occlusion occurred. Each vial of microspheres was diluted up to 20 mL with a mixture of 50/50 contrast and saline. Most interventions were performed as a same-day outpatient procedure under local anesthesia with or without moderate sedation.

### Outcome Measures

The primary outcome measure was the 12 month change in the International Prostate Symptom Score (IPSS). Secondary outcome measures included: (1) IPSS measurements at 3 and 24 months; (2) device-related and procedure-related AEs at 3, 12, and 24 months following PAE; (3) technical success (i.e., technically successful embolization of at least one prostatic artery); (4) removal of the indwelling catheter in those treated for AUR; (5) number of patients with refractory or recurrent LUTS due to BPH at 3, 12, and 24 months post-PAE; and (6) 12 month changes in erectile function, assessed using the Sexual Health Inventory for Men (SHIM) questionnaire.

Clinical characteristics assessed included prostate size, maximum urinary flow rate (Qmax), post-void residual (PVR) volume, and prescribed prostate medications. Procedural characteristics assessed included volume of embolic administered, unilateral versus bilateral embolization, and procedure time.

Treatment-related AEs were reviewed and adjudicated based on the clinical judgement of an independent physician with experience in PAE. AEs were considered to be serious if they met any of the following criteria: (1) resulted in death; (2) were life-threatening; (3) required in-patient hospitalization or prolongation of existing hospitalization; (4) resulted in persistent or significant disability/incapacity; (5) were considered an important medical event, which was defined as an event that may not result in death, be life-threatening, or necessitate hospitalization but, based on discretion of the medical staff, may jeopardize the patient and/or necessitate medical or surgical intervention.

### Statistical Analysis

Continuous data were summarized using mean and standard deviation. Categorical variables were summarized using frequency, counts, and percentages. Technical parameters were reported as the proportion of patients that underwent unilateral or bilateral PAE. Relative changes in IPSS and quality of life (QoL) over the follow-up period were evaluated using independent t-tests and reported as mean changes with standard deviations. Changes in SHIM from baseline to 12 months following PAE were reported as means and standard deviations; *p* values < 0.05 were considered significant.

## Results

### Patient Demographics and Procedural Characteristics

**Fig. 1:**
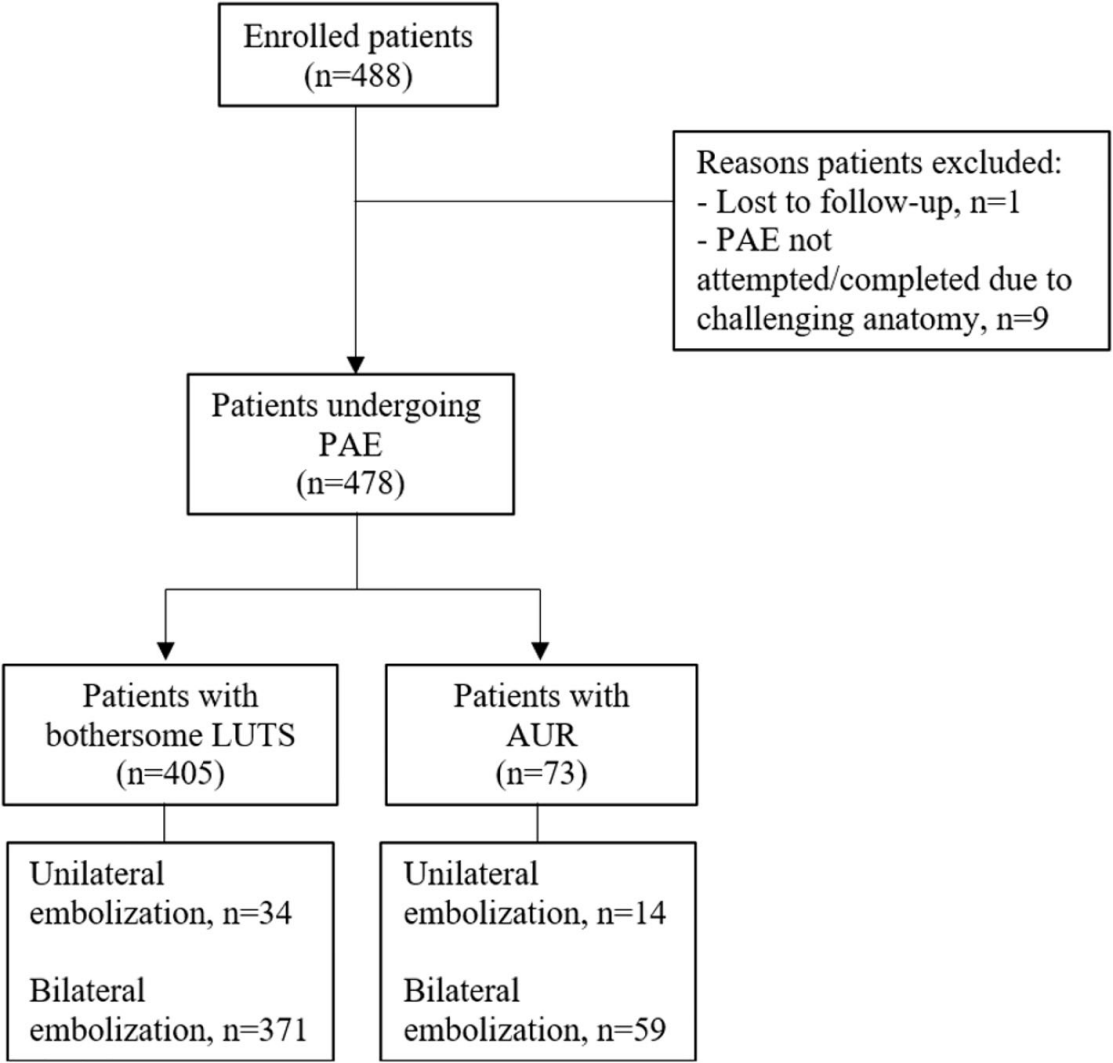
Patient flow diagram AUR, acute urinary retention; LUTS,  lower urinary tract symptoms; PAE, prostatic artery embolization

Of the 488 patients enrolled, 10 were excluded from analysis due to lost to follow up (n = 1) and PAE not attempted/completed due to challenging anatomy (n = 9) (Fig. 1). The mean age of all patients was 70 ± 8 years; patients in the LUTS cohort were significantly younger than patients in the AUR cohort (69 ± 8 vs. 75 ± 10 years, *p *< 0.0001).

Of the 478 patients that underwent PAE, 405 (84.7%) were for bothersome LUTS and 73 (15.3%) were for AUR (Fig. 1). The mean volume of diluted microspheres administered was 13.0 ± 6.9 mL (LUTS cohort: 13.0 ± 7.0; AUR cohort: 12.6 ± 6.6). The mean PAE procedure time was 111.1 ± 45.3 min overall (LUTS cohort: 109.5 ± 45.5; AUR cohort: 120.1 ± 44.2).

### Primary Outcome Measure

**Fig. 2:**
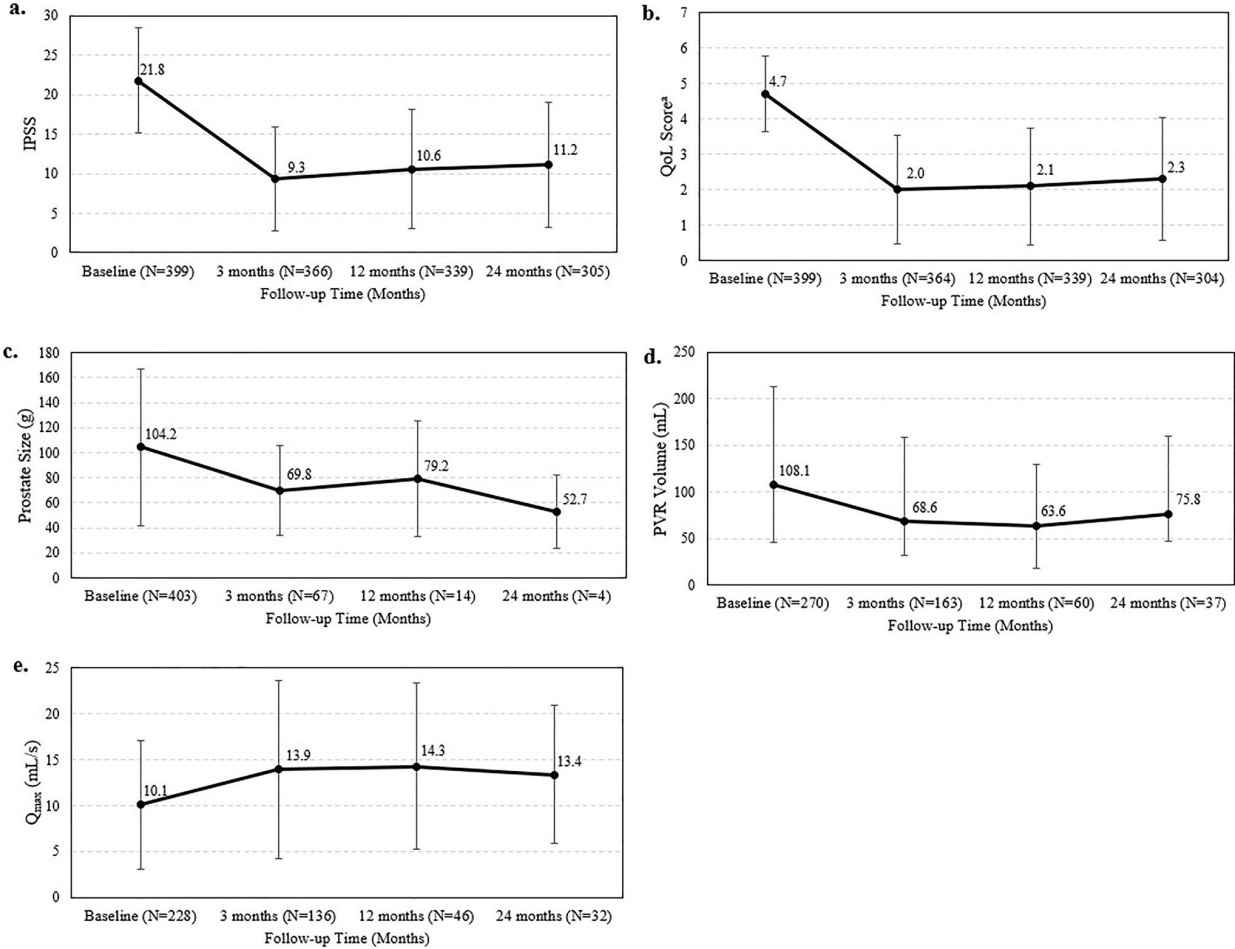
Changes in IPSS, QoL^a^, and clinical characteristics of patients with bothersome LUTS. IPSS,  International Prostate Symptom Score; LUTS, lower urinary tract symptoms; PVR, post-void residual; Qmax, maximum urinary flow rate; QoL, quality of life; SHIM, Sexual Health Inventory for Men ^a^QoL score is based on a single question within the IPSS assessment that asks patients “If you were to spend the rest of your life with your urinary condition just the way it is now, how would you feel about that?”, scores are graded based on the following 0 (delighted), 1 (pleased), 2 (mostly satisfied), 3 (mixed about equally satisfied and dissatisfied), 5 (mostly dissatisfied), and 6 (terrible)

Among the 405 patients with bothersome LUTS, the 12 month relative change in IPSS was − 11.1 ± 8.3 (*p* < 0.001; Table 1). The mean IPSS at baseline was 21.8 ± 6.6 and 12 months post-PAE, the mean IPSS was 10.6 ± 7.5 (Table 1; Fig. 2). For patients in the AUR cohort, the mean IPSS was 7.7 ± 5.3; as no baseline IPSS data were available for the AUR cohort, the relative change could not be calculated.

**Table 1:** Summary of IPSS

Patients with bothersome LUTS (N = 405)					
		Baseline	3 month	12 month	24 month
IPSS	N	399	366	339	305
	Mean score (SD)	21.8 (6.6)	9.3 (6.6)	10.6 (7.5)	11.2 (7.9)
	Relative change (SD)	–	− 12.4 (7.8)	− 11.1 (8.3)	− 10.2 (8.5)
	*p* value	–	< 0.001	< 0.001	< 0.001
SHIM	N	395	–	325	–
	Mean score (SD)	13.8 (8.5)	–	13.9 (8.8)	–
	Relative change (SD)	–	–	− 0.04 (6.5)	–
	*p* value	–	–	0.912	–
QoL^a^	N	399	364	339	304
	Mean score (SD)	4.7 (1.1)	2.0 (1.5)	2.1 (1.7)	2.3 (1.7)
	Relative change (SD)	–	− 2.7 (1.7)	− 2.6 (1.7)	− 2.3 (1.8)
	*p* value	–	< 0.001	< 0.001	< 0.001
Prostate size (g)	N	403	67	14	4
	Mean size (SD)	104.2 (63.0)	69.8 (36.3)	79.2 (46.1)	52.7 (29.1)
	Relative change (SD)	–	− 28.8 (32.7)	− 6.7 (30.6)	− 20.8 (33.5)
	*p* value	–	< 0.001	0.428	0.304
PVR volume (mL)	N	270	163	60	37
	Mean volume (SD)	108.1 (104.7)	68.6 (89.4)	63.6 (65.2)	75.8 (84.6)
	Relative change (SD)	–	− 34.5 (104.30)	− 40.4 (86.8)	− 34.5 (81.6)
	*p* value	–	< 0.001	< 0.001	0.015
Qmax (mL/s)	N	228	136	46	32
	Mean (SD)	10.1 (7.0)	13.9 (9.6)	14.3 (9.0)	13.4 (7.5)
	Relative change (SD)	–	4.9 (10.6)	5.3 (9.3)	2.4 (10.4)
	*p* value	–	< 0.001	< 0.001	0.197

QoL^a^, SHIM, and clinical characteristics over the follow-up period among patients with bothersome LUTS

IPSS, International Prostate Symptom Score; LUTS, lower urinary tract symptoms; PVR, post-void residual; Qmax, maximum urinary flow rate; QoL, quality of life; SHIM, Sexual Health Inventory for Men; SD, standard deviation; –, data not available or unable to be calculated (e.g., insufficient data, no baseline scores available [for IPSS and QoL])

^a^QoL score is based on a single question within the IPSS assessment that asks patients “If you were to spend the rest of your life with your urinary condition just the way it is now, how would you feel about that?”, scores are graded based on the following 0 (delighted), 1 (pleased), 2 (mostly satisfied), 3 (mixed about equally satisfied and dissatisfied), 5 (mostly dissatisfied), and 6 (terrible)

### Secondary Outcome Measures

Technical success was achieved in all patients (Fig. 1). In both cohorts, most patients underwent bilateral embolization (LUTS cohort: 91.6%; AUR cohort: 80.8%). Among patients in the LUTS cohort, 34 (8.4%) underwent unilateral embolization compared to 14 (19.2%) patients in the AUR cohort.

In the bothersome LUTS cohort, the mean IPSS at 3 months was 9.3 ± 6.6 and the mean IPSS at 24 months was 11.2 ± 7.9. The relative changes in IPSS at 3 and 24 months post-PAE were − 12.4 ± 7.8 and − 10.2 ± 8.5, respectively, for the LUTS cohort (*p* < 0.001 for both time points). The mean SHIM scores at baseline and 12 months post-PAE were 13.8 ± 8.5 and 13.9 ± 8.8, respectively (relative change: − 0.04 ± 6.5; *p* = 0.912). The relative changes in QoL at 3, 12, and 24 months post-PAE were − 2.7 ± 1.7, − 2.6 ± 1.7, and − 2.3 ± 1.8, respectively (all *p* < 0.001). The relative change in prostate size was − 28.8 ± 32.7g at 3 months (*p* < 0.001), − 6.7 ± 30.6 g at 12 months (*p* = 0.428), and − 20.8 ± 33.5 g at 24 months (*p* = 0.304). The relative change in PVR volume was − 34.5 ± 104.3 mL at 3 months (*p* < 0.001), − 40.4 ± 86.8 mL at 12 months (*p* < 0.001), and − 34.5 ± 81.6 mL at 24 months (*p* = 0.015). The relative changes in Qmax were 4.9 ± 10.6 mL/s at 3 months (*p* < 0.001), 5.3 ± 9.3 mL/s at 12 months (*p* < 0.001), and 2.4 ± 10.4 mL/s at 24 months (*p* = 0.197). The mean values for clinical characteristics across the 24 month follow-up period are provided in Table 1.

Of the 73 patients in the AUR cohort, 48 patients (65.8%) had their indwelling bladder catheter removed within 3 months following PAE and remained catheter free during the study. The mean SHIM scores at baseline and 12 months post-PAE for the AUR cohort were 11.2 ± 10.0 and 9.8 ± 8.7, respectively (relative change: − 1.4 ± 7.8; *p* = 0.431). The relative change in prostate size was − 10.6 ± 21.8 g at 3 months (*p* = 0.339) and − 6.6 ± 39.1 g at 12 months (*p* = 0.727). The relative change in PVR volume was 10.8 ± 53.5 mL at 3 months (*p* = 0.715).

Of the 478 patients in the study, 16 (3.3%) underwent re-embolization following the initial PAE procedure due to secondary clinical failure. Of these 16 patients, 12 with bothersome LUTS underwent re-embolization within 24 months (n = 8 within 12 months, n = 4 within 24 months) of the initial procedure and 4 patients with AUR at baseline underwent re-embolization within 12 months of the initial procedure (none within 24 months). Twenty-four months post-PAE, 34.7% (17/49) of patients with AUR were still using BPH medications (n = 7: α-blockers only; n = 4: 5-α reductase inhibitors only; n = 6: ≥ 2 combined medications), and 34.3% (106/309) with bothersome LUTS (n = 69 α-blockers only; n = 9 5-α reductase inhibitors only; n = 28 ≥ 2 combined medications). Over the 24 month follow-up period, 32 of the 478 patients (6.7%) underwent surgery or a minimally invasive surgical therapy after 14.5 ± 9.3 months (n = 26 within 12 months, n = 6 within 24 months). Of these 32 patients, 24 were from the LUTS cohort (n = 19 at 12 months, n = 5 at 24 months) and 8 were from the AUR cohort (n = 7 at 12 months, n = 1 at 24 months).

### Adverse Events

A total of 55 out of 478 (11.5%) patients had ≥ 1 AE (Table 2), with 10 (2.1%) experiencing a serious AE (Table 3). The most common AE was self-limiting irritative symptoms (21.5%; 103/478) (Table 2). One patient experienced a penile ulceration that resolved. One patient developed a rectoprostatic fistula following PAE for bothersome LUTS, this patient had a prior history of prostate cancer and underwent radiation therapy prior to PAE. The patient underwent conservative medical management for the fistula.

**Table 2:** Summary of adverse events

Adjudicated adverse events	Patients affected/at risk (%)^a^	Total no. of events
	55/478 (11.5%)	205
*Gastrointestinal disorders*		
Abdominal spasm	5/478 (1.0%)	6
Blood in stool	3/478 (0.6%)	3
Bowel/Fecal incontinence	2/478 (0.4%)	2
Constipation	3/478 (0.6%)	3
*General disorders and administration site conditions*		
Fatigue	20/478 (4.2%)	20
Fever	1/478 (0.2%)	1
Low grade fever	1/478 (0.2%)	1
*Infections and infestations*		
Urinary tract infection	1/478 (0.2%)	1
*Injury, poisoning, and procedural complications*		
Post-procedural pain	7/478 (1.5%)	7
*Nervous system disorders*		
Burning sensation	2/478 (0.4%)	2
*Renal and urinary disorders*		
Acute urinary retention requiring catheterization	10/478 (2.1%)	10
Hematuria	14/478 (2.9%)	14
Self-limiting irritative symptoms^b^	103/478 (21.5%)	108
*Reproductive system and disorders*		
Bloody semen	11/478 (2.3%)	11
Pelvic pain	13/478 (2.7%)	13
Penile pain	1/478 (0.2%)	2
*Skin and subcutaneous tissue disorders*		
Penile ulceration	1/478 (0.2%)	1

^a^Patients could experience more than one adverse event

^b^Self-limiting irritative symptoms included: bladder spasm, n = 6; burning micturition, n = 29; contracted bladder, n = 1; frequency urinary, n = 12; incontinence urinary, n = 9; painful urination, n = 31; urgency urination, n = 10; urinary frequency, n = 3; urination difficulty, n = 2

**Table 3:** Summary of serious adverse events

Adjudicated serious adverse event	Patients affected/at risk (%)	Total no. of events	Site-reported outcome
	10/478 (2.1%)	10	
Acute renal failure	1/478 (0.2%)	1	Ongoing
Dizziness upon walking	1/478 (0.2%)	1	Recovered
Dyspnea and congestive heart failure	1/478 (0.2%)	1	Recovered
Emergency admission with abdominal pain and vomiting	1/478 (0.2%)	1	Recovered
Pseudo aneurysm at point of puncture	1/478 (0.2%)	1	Recovered
Hypertension	1/478 (0.2%)	1	Recovered
Prostatitis	1/478 (0.2%)	1	Recovered
Rectoprostatic fistula	1/478 (0.2%)	1	Ongoing
Urinary tract infection and hypotension	1/478 (0.2%)	1	Recovered
Urinary tract infection with hypotension and urinary retention six days after embolization	1/478 (0.2%)	1	Recovered

## Discussion

Across all timepoints evaluated in this study, the mean changes in IPSS and QoL scores suggest durability of the clinical benefits associated with PAE. Additionally, the low rates of AEs and serious AEs observed during the follow-up period suggest that PAE is a safe option to address the clinical needs of this patient population.

The significant changes in IPSS and QoL scores relative to baseline among patients in this study align with prior studies [11, 14, 15]. In an observational study of men with LUTS [14], the differences in IPSS and QoL 12 months following PAE relative to baseline were − 10.9 and − 2.6, respectively, which match the − 11.1 and − 2.6 changes in IPSS and QoL scores, respectively, that were observed in the present study.

Definitions of clinical success following PAE vary across studies [4, 17, 30]. A commonly used definition for patients with bothersome LUTS is an IPSS of ≤ 15 and/or a ≥ 25% decrease in IPSS relative to baseline [17, 30]; for patients with AUR, clinical success is defined as the removal of the indwelling catheter. In this study, clinical success was not determined a priori. However, in the LUTS cohort 79.3% (242/305) of patients had an IPSS of ≤ 15 or a ≥ 25% decrease in IPSS at 24 months. This aligns with prior studies reporting clinical success rates of 82–90%. A total of 65.8% (48/73) of patients with AUR were able to have their indwelling catheter removed within 3 months of embolization and remained catheter free for the remainder of the 24 month follow-up period. This finding is slightly lower than other studies [16, 21] that reported indwelling catheter removal in 73–75% of patients following PAE. However, due to the small sample sizes in prior studies (n = 20 [21] and n = 26 [16]), the proportion of patients in this study that were catheter-free following embolization may be comparable.

In the present study, 16 (3.3%) patients were re-embolized within 24 months, 32 (6.7%) underwent surgery or another minimally invasive procedure. Approximately one-third of patients in both cohorts were still using medications to manage their BPH, this proportion aligns with a prior study that reported 31% of patients using BPH medications following PAE [31]. Although PAE is known to be an effective therapy for BPH with LUTS, it is not uncommon for some patients to require additional treatment due to recurrence of symptoms or inadequate response to the initial PAE procedure [14, 17, 32, 33]. Despite some variation in the proportion of patients that require re-intervention or continued medication, PAE is still considered an effective therapy with a durable response in most patients.

PAE is considered a safe procedure; serious complications are rare [34]. One study [4] reported a major complication rate of 1.6%, which aligns with the 2.1% reported in the present study. In this study, the occurrence of non-target embolization was low: one patient experienced penile ulceration, which was reversible, and aligns with prior reports of low rates of non-target embolization [4, 14].

In this study, there was a noticeable difference in the proportion of patients that underwent unilateral embolization (8% in the LUTS cohort vs. 19% in the AUR cohort). A potential reason for this discrepancy may be the significant difference in age as older patients have more atheromatous arteries that can sometimes preclude access to the prostatic artery. Nevertheless, the proportion of patients in both cohorts that experienced technical success of the procedure was high and aligns with prior evidence demonstrating the technical feasibility of the procedure and its clinical utility [35, 36]. Moreover, the clinical benefits and tolerable safety profile associated with PAE have been recognized by the Society for Interventional Radiology and the American Urological Association [37, 38]. As additional evidence demonstrating the tolerable safety and efficacy profiles associated with PAE emerge, future studies evaluating what, if any, impact heterogenous techniques have on outcomes will be an important consideration to address.

This study should be considered within the context of certain limitations. First, missing patient data due to patients lost to follow up (e.g., missed appointments) resulted in the inability to evaluate data for every patient across all timepoints. Second, without a control cohort, comparisons could not be made regarding the magnitude of improvement for untreated patients, or patients treated with other surgical procedures. Third, although 478 patients is an acceptable sample size for real-world data, no hypothesis testing was performed across all timepoints for both cohorts; therefore, we are unable to confirm whether the mean changes in all study measures relative to baseline were significant. Finally, this study was unable to capture the full range of reasons for subsequent procedures or reasons for loss of follow-up.

In conclusion, findings from this multicenter, international, prospective, cohort study of patients with BPH and bothersome LUTS, or AUR, who underwent PAE provide further evidence supporting the clinical utility of PAE. The generally low AE rates may encourage broader use in these patients.

## Data Availability

Reasonable requests for anonymized data that support the findings of this work can be made to the corresponding author.

## Declarations

### Previous Presentation

A synopsis of a portion of the study results was presented at the 2022 Global Embolization and Oncology Symposium Technologies (GEST) conference.
